# A Novel Bromophenol Compound from *Leathesia nana* Inhibits Breast Cancer in a Direct Tumor Killing and Immunotherapy Manner

**DOI:** 10.3390/molecules28145349

**Published:** 2023-07-12

**Authors:** Ruochen Sun, Mi Zhang, Bufan Li, Shan Jiang, Wanpeng Yu, Lina Yang, Yantao Han, Zhangfeng Zhong, Wenwen Zhao

**Affiliations:** 1 College of Basic Medical Sciences, Qingdao University, 308 Ningxia Road, Qingdao 266021, China; sunruochen233@163.com (R.S.); 17862846170@163.com (M.Z.); lbf83665415@163.com (B.L.); lhy971207@163.com (W.Y.); lanyu-323@163.com (L.Y.); daisy@qdu.edu.cn (Y.H.); 2 Affiliated Hospital of Integrated Traditional Chinese and Western Medicine, Nanjing University of Chinese Medicine, Nanjing 210028, China; 18254887323@163.com; 3 Laboratory of Translational Medicine, Jiangsu Province Academy of Traditional Chinese Medicine, Nanjing 210028, China; 4 Macao Centre for Research and Development in Chinese Medicine, State Key Laboratory of Quality Research in Chinese Medicine, Institute of Chinese Medical Sciences, University of Macau, Macao SAR 999078, China

**Keywords:** bromophenol compound, ferroptosis, immunotherapy, breast cancer, cardiotoxicity

## Abstract

Considering the resistance and toxicity of traditional chemotherapeutic drugs, seeking potential candidate for treating breast cancer effectively is a clinical problem that should be solved urgently. Natural products have attracted extensive attention, owing to their multi-target advantages and low toxicity. In the current study, the effects of XK-81, a novel bromophenol compound extracted from *Leathesia nana*, on breast cancer, and its underlying mechanisms, were explored. Firstly, data from in vitro experiments indicated that 4T-1, one of common mouse breast cancer cell lines, was a XK-81-susceptible cell line, and ferroptosis was the major death manner in response to XK-81 treatment, which was evidenced by increasing intracellular Fe^2+^ and ROS level with condensed mitochondrial membrane densities, as well as decreasing the protein expressions of SLC7A11 and GPX4. In vivo, XK-81 suppressed the growth of 4T-1 breast-tumor in both BALB/C mice and zebrafish. Obviously, XK-81 decreased the protein expression of SLC7A11 and GPX4 in tumor tissues, hinting at the occurrence of ferroptosis. Moreover, XK-81 increased CD8+ T cells and NK cells numbers and regulated M1/M2 macrophage ratio in tumor tissues, indicating XK-81’s immunotherapeutic effect. Additionally, the secretions of immune-related cytokines, including TNF-α, IL-1β, and IL-12, were elevated with XK-81 stimulation in RAW 264.7 cells. Intriguingly, compared with doxorubicin-induced heart damage, XK-81 demonstrated the therapeutic advantage of little cardiotoxicity on the heart. XK-81 demonstrated potential antitumor advantage by both directly inducing ferroptosis-mediated death of tumor cells and immunization.

## 1. Introduction

The latest global cancer burden data released by the World Health Organization’s IARC in 2020 shows that breast cancer has officially overtaken lung cancer as the world’s leading cancer, with 2.26 million new cancers compared to 2.2 million for lung cancer [1,2]. With the duration of drug application being longer, more and more tumor cells escape from apoptosis and develop chemotherapeutic resistance, hinting at the existence of other death manners for tumors [3,4,5,6]. Therefore, novel efficacious therapeutic concepts and avenues are urgently needed.

Increasing preclinical evidence suggests that ferroptosis may be a therapeutic strategy to reduce resistance to chemotherapeutic drugs [7,8]. Ferroptosis has been observed in the therapeutic process of clinical first-line anticancer drugs [9]. When there are NSCLC cell lines in treatment, Cisplatin binds with glutathione to form a PT-GS complex, resulting in peroxidase 4 (GPX4) inactivation and leading to ferroptosis [10]. The combination of Lapatinib and Siramesine could promote ferroptosis in breast cancer MDA-MB-231 and MCF-7 cells, which did not occur when they were used alone [11]. Nowadays, more and more natural products have been revealed to induce ferroptosis. Dihydroartemisinin promotes cysteine deprivation and GPX4 inhibition and then induces the ferroptosis of HepG2 hepatoma cells [12]. Erianin from dendrobium exhibits high efficiency, low toxicity, and anticancer activity by inducing calcium-dependent ferroptosis [13]. Therefore, ferroptosis is becoming a new strategy for cancer treatment.

Recently, immunotherapy has become an innovative treatment for tumors [14,15,16,17,18]. It reawakens immune cells to overcome immune escape and finally eliminates cancer cells [19,20,21,22]. Generally, tumor cells undergoing ferroptosis can release injury signals to regulate immune activity and function, and the immune system further releases cytokines to regulate the sensitivity of tumor cells to ferroptosis [23,24,25,26,27]. Therefore, the combination of chemotherapy with immunotherapy will produce a preferable therapeutic effect [26,28,29]. Combination therapy not only kills tumor cells directly, but it also prevents tumor immune escape to further enhance an anticancer effect.

Due to the complexity and biodiversity of the ocean, many compounds from the ocean have excellent anti-inflammatory, anti-tumor, and cardiovascular disease protective effects, with few side effects [30,31,32]. Bromophenol compounds are natural compounds derived only from the ocean [33,34]. A variety of studies have reported that they have splendid antibacterial and anti-tumor activity [35,36], while the mechanisms are poorly explored [37].

In this study, a novel bromophenol compound extracted from *Leathesia nana*, named XK-81, was studied. Although XK-81 was supposed to have anti-tumor potentialities, its exact pharmacological activity remains unclear. In the present study, the effect of XK-81 on tumors was explored on both BALB/C mice and xenograft zebrafish model. Then, its mechanisms were further revealed, based on cell models.

## 2. Results and Discussion

### 2.1. XK-81 Selectively Inhibited 4T-1 Cell Proliferation In Vitro

**Figure 1:**
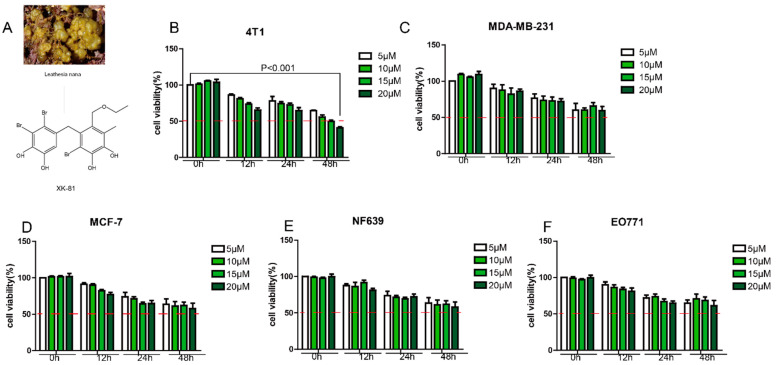
The inhibitory effects of XK-81 against breast cancer in vitro. (**A**) The origin and chemical structure of XK-81. (**B**) Mouse breast cancer 4T-1 cells. (**C**) Human breast cancer MDA-MB-231 cells. (**D**) MCF-7 cells, mouse breast cancer. (**E**) NF639 cells and (**F**) EO771 cells were treated with XK-81 at the indicated concentrations (5 µM, 10 µM, 15 µM, and 20 µM) for 0 h, 12 h, 24 h, and 48 h, and cell viability was detected by a MTT assay. XK-81, *Leathesia nana* bromophenol.

The origin and chemical structure of XK-81 is shown in Figure 1A. The methylthiazole tetrazolium (MTT) assay was used to measure the cytotoxic effects of XK-81 on mouse breast cancer cells 4T-1, NF639, and EO771, as well as human breast cancer cells MDA-MD-231 and MCF-7 at a series of concentrations (5, 10, 15 and 20 µM) and times (0 h, 12 h, 24 h, and 48 h). Our results showed that when the dose of XK-81 increases, the cell viability of a series of breast cancer cell decreased. Obviously, the survival rates of XK-81 (20 µM) were higher than 50% on most breast cancer cells, including MDA-MB-231 (61.23%), MCF-7 (55.32%), NF639 (54.81%), and EO771 (65.45%) (Figure 1B–F). While XK-81 has strong cytotoxicity and sensitivity towards 4T-1 cells (Figure 1B), with a survival rate of 45.35% hinting at XK-81’s therapeutic advantage against 4T-1 cells with an optimal dose 20 µM and an optimal time 48 h.

### 2.2. XK-81 Treatment Inhibited Tumor Growth In Vivo

**Figure 2:**
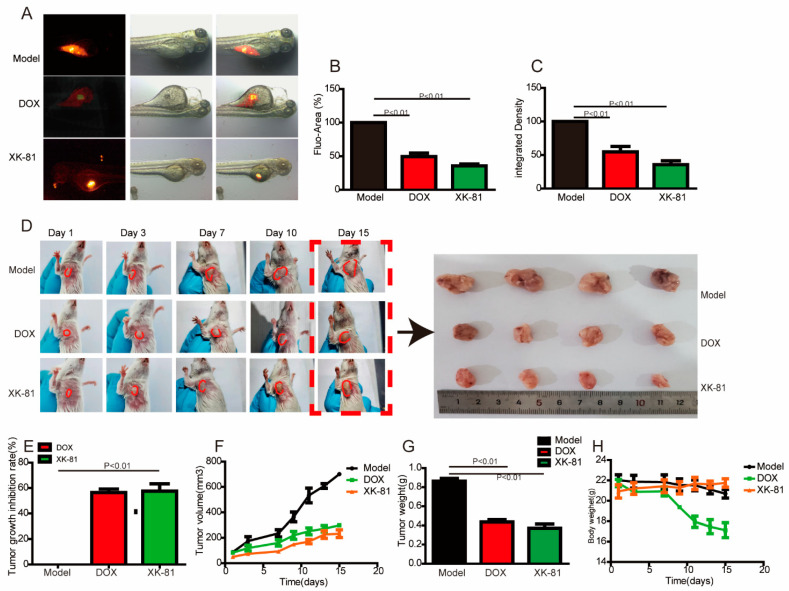
The inhibitory effects of XK-81 on tumor growth were determined. In the xenograft zebrafish model, (**A**) the tumor was indicated by red fluorescence. The percentage of (**B**) fluorescence area and (**C**) fluorescence intensity was processed by ImageJ. In the subcutaneous 4T-1 breast tumor mouse model, (**D**) photographs of the tumors harvested on day 15 are presented. (**E**) The tumor growth inhibition rate. (**F**) The tumor volume growth curves until day 15. (**G**) The tumor weight on day 15. (**H**) Changes in the body weight of mice. Values are presented as mean ± SD, n = 5. DOX, doxorubicin; XK-81, *Leathesia nana* bromophenol.

To further verify the data from in vitro experiments, a xenograft zebrafish model and subcutaneous 4T-1 breast tumor mouse model were established to evaluate the antitumor effects of XK-81 in vivo. Firstly, in the xenograft zebrafish model, injected 4T-1 cells labeled with CM-Dil were rapidly proliferated and identified with a strong red fluorescence signal. After XK-81 treatment, the fluorescence area was immediately reduced to nearly 50%, and the fluorescence intensity was weakened to 63.49%, confirming XK-81’s antitumor effect. (Figure 2A–C). In a subsequent study, a subcutaneous 4T-1 breast tumor mouse model was constructed to evaluate the antitumor effect of XK-81. When the tumor grew to ≈50 mm^3^, mice were divided into three groups randomly, and they were administered with 50 mg/kg intraperitoneal XK-81 (dissolved in 0.5% carboxymethyl cellulose-Na), as well as 3 mg/kg tail intravenous DOX for 14 days. Compared to the rapid growth of tumor tissue in the Model group, both XK-81 and DOX visibly impeded tumor growth, and the tumor growth inhibition rates are 57.34% for DOX and 59.56% for XK-81. XK-81 and DOX visibly impeded tumor growth compared to the rapid growth of the Model group (Figure 2D–G). Compared with the DOX group, the treatment of XK-81 did not introduce a loss of body weight, indicating lower acute toxicity than DOX (Figure 2H).

### 2.3. XK-81 Had Little Cardiotoxicity in Tumor-Bearing Mice

**Figure 3:**
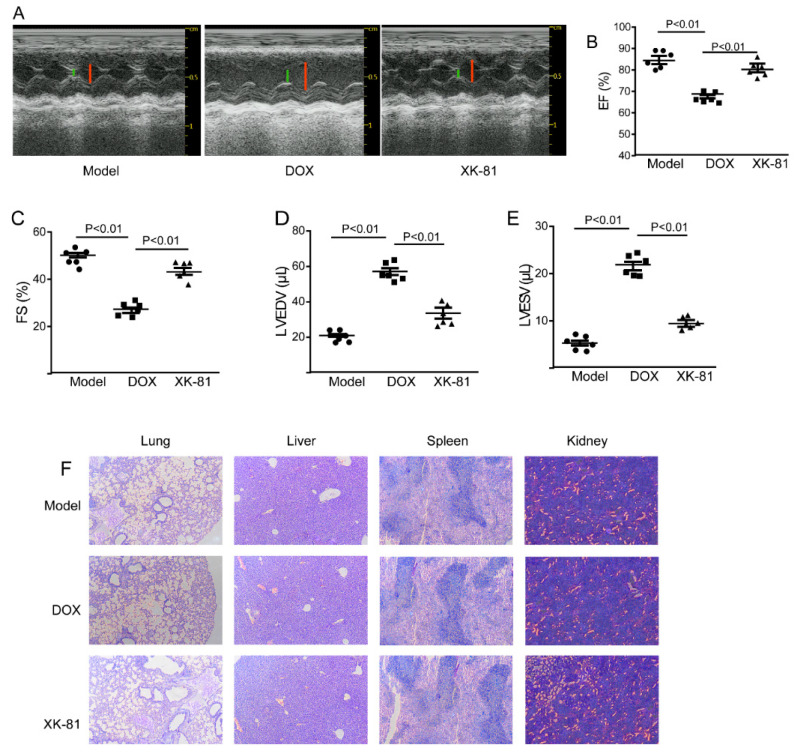
The protective effect of XK-81 on tumor-bearing mice. (**A**) Representative images of echocardiographs and the green and red lines represent left ventricular end-systolic and end-diastolic volumes, respectively. Cardiac function evaluation indexes (**B**) EF, (**C**) FS, (**D**) LVEDV, and (**E**) LVESV were detected by transthoracic echocardiography. (**F**) Non-target organs, including the liver, spleen, kidney, and lung were dyed by HE staining. DOX, doxorubicin; EF, ejection fraction; FS, fractional shortening; LVEDV, left ventricular end-diastolic volume; LVESV, left ventricular end-systolic volume; XK-81, *Leathesia nana* bromophenol.

Considering that cardiotoxicity is the most severe complication accompanied by anticancer drug of chemical therapy, cardiotoxicity of XK-81 in mice was assessed by echocardiographs. As shown in Figure 3A–E, after 14 days of treatment with DOX in breast cancer mouse models, cardiac function in tumor-bearing mice was prominently impaired, evidenced by decreasing ejection fraction (EF) (from 85.32% in the model group to 69.37% in the DOX group) and fractional shortening (FS) (from 52.27% in the model group to 21.83% in the DOX group), while increasing left ventricular end-systolic volume (LVESV) (from 20.12% in model group to 59.37% in DOX group) and left ventricular end-diastolic volume (LVEDV) (from 5.11% in model group to 22.32% in DOX group). These data indicated the pathological changes in the DOX group in terms of the structure and function of the left ventricle. Meanwhile, after 14 days of treatment with XK-81, all the above indexes, including EF, FS LVESV, and LVEDV, were obviously improved and close to the model group. These data suggested that XK-81 had little cardiotoxicity on mice, demonstrating its therapeutic advantages. Moreover, XK-81 has no cytotoxicity on non-target organs, including the liver, spleen, kidney, and lung (Figure 3F). Collectively, we provided direct evidence for the safety of XK-81.

### 2.4. XK-81 Promoted 4T-1 Cells Death via Ferroptosis 

**Figure 4:**
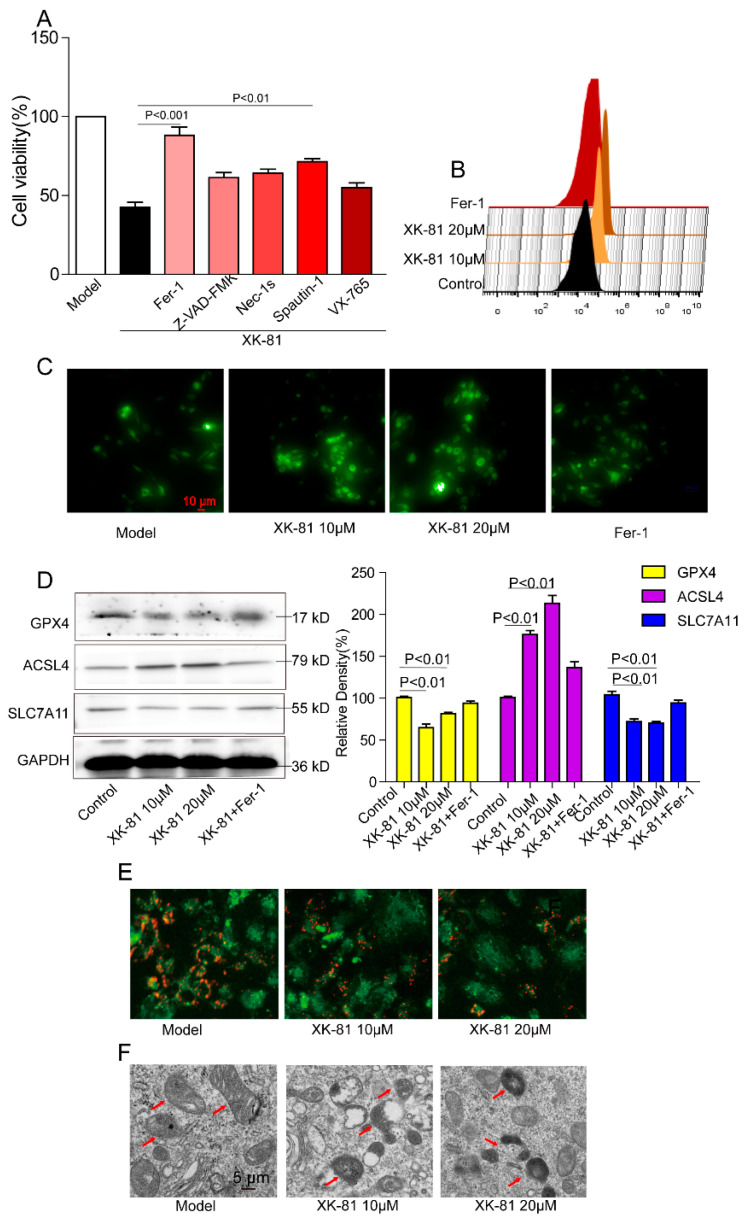
The effect of XK-81 on 4T-1 cells’ death and related mechanisms in vitro. 4T-1 cells were pretreated with Fer-1(10 µM), Z-VAD-FMK (1 µM), Nec-1s (10 µM), Spautin-1 (10 µM), and VX-765 (1 µM) for 1 h and then stimulated with XK-81 (20 µM, 48 h). (**A**) Cell viability was detected with a MTT assay. (**B**) Fluorometric analysis of lipid ROS was carried out using C11-BODIPY 581/591 dye. (**C**) Intracellular Fe^2+^ was assessed with the dye Ferro Green. (**D**) ACSL4, SLC7A11, and GPX4 were detected by Western blotting. (**E**) JC-1 dye was applied to monitor mitochondrial health and (**F**) electron microscopy was used to observe morphological changes of mitochondria and the red arrows represented mitochondria. ACSL4, long-chain acyl-CoA synthetase 4; Fer-1, ferroptosis inhibitor; GPX4, glutathione peroxidase 4; XK-81, *Leathesia nana* bromophenol; Nec-1s, necrotic inhibitor; Spautin-1, autophagy inhibitor; VX-765, pyroptosis inhibitor; Z-VAD-FMK, apoptosis inhibitor.

In the following experiments, exact death manners and related mechanisms for XK-81 inducing 4T-1 cells death were explored. As shown in Figure 4A, XK-81 killed more than half of the 4T-1 cells. Compared with apoptosis inhibitor Z-VAD-FMK, necrotic inhibitor Nec-1s, autophagy inhibitor Spautin-1, and pyroptosis inhibitor VX-765, noticeably, only ferroptosis inhibitor Fer-1 restored 4T-1 cells viability to 92.13%, implying the occurrence of ferroptosis for XK-81-induced 4T-1 death (Figure 4A). Subsequently, a series of ferroptosis indexes, including iron overload and lipid peroxidation, as well as ferroptosis-related protein long-chain acyl-CoA synthetase 4 (ACSL4) and glutathione peroxidase 4 (GPX4), were detected, respectively, to further demonstrate the ferroptosis characteristics in 4T-1 cells. Data from flow cytometry showed a rightward shift of the peak top from the control group to the XK-81 group, hinting at a large amount of lipid ROS generation (Figure 4B). As shown in Figure 4C, cellular Fe^2+^, accompanied by green fluorescence, was clearly observed in the XK-81 group, while Fer-1 significantly reduced fluorescence intensity. Meanwhile, the decrease in GPX4/SLC7A11 and the increase in ACSL4 protein expression levels were observed in XK-81-treated cells, while mitochondrial membrane potential hyperpolarization (Figure 4E), swollen mitochondria, and the disappearance of mitochondrial cristae (Figure 4F) occurred in XK-81-irritated cells. All the results hinted at the occurrence of ferroptosis in 4T-1 cells in response to XK-81.

### 2.5. XK-81 Promoted Reactive Oxygen Species (ROS) Generation in 4T-1 Cells 

**Figure 5:**
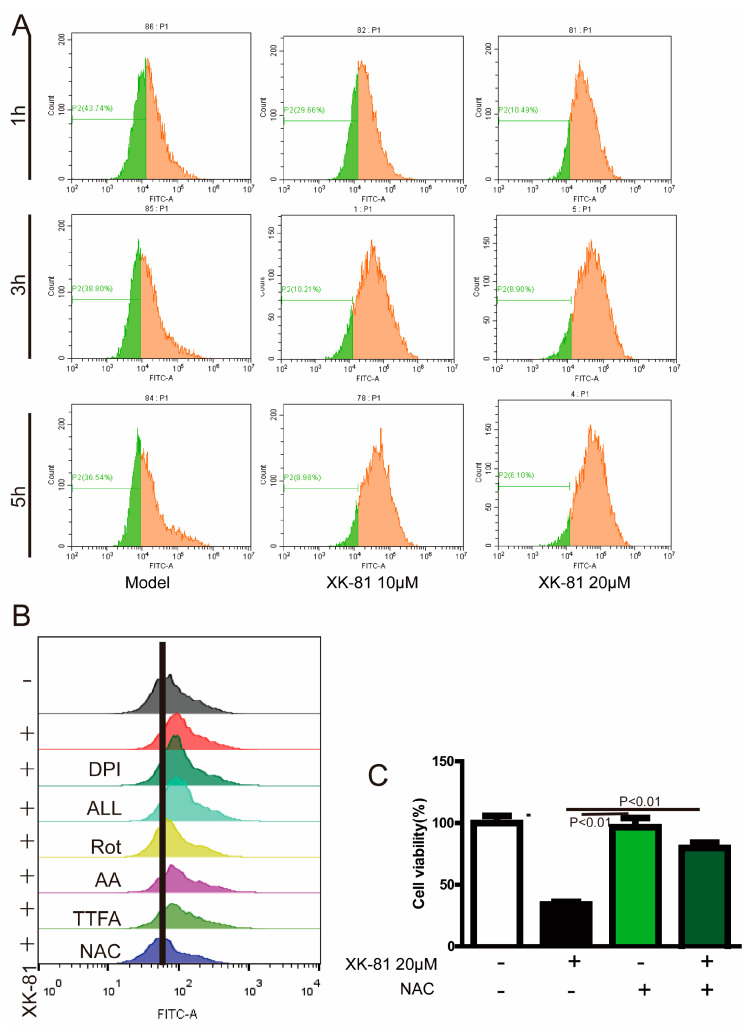
XK-81 increased ROS generation in response to 4T-1 cells death. Cells were treated with XK-81 (0, 10, and 20 µM) at 1, 3, and 5 h, (**A**) ROS generation was detected with the probe DCFH-DA by flow cytometry. (**B**) The cells were pretreated with Rot (10 µM), TTFA (10 µM), AA (100 nM), ALL (5 µM), DPI (100 nM), and NAC (5 mM) separately for 1 h and then stimulated with XK-81 (20 µM, 48 h), and then cellular ROS was determined by flow cytometry with DCFH-DA dye. (**C**) 4T-1 cells were pretreated with NAC (5 mM) for 1 h and then stimulated with XK-81 (20 µM) for 48 h, and cell viability was detected with a MTT assay. Rot, rotenone; AA, antimycin A; ALL, allopurinol; DCFH-DA, 2′-7′-dichlorofluorescein diacetate; DPI, diphenyleneiodonium chloride; XK-81, *Leathesia nana* bromophenol; NAC, N-acetyl-L-cysteine; TTFA, thenoyltrifluoroacetone.

Previous studies have shown that the imbalance between ROS production and consumption plays a momentous role in the development and progression of breast cancer [38]. In the study, compared with untreated 4T-1 cells, there occurred a ROS-induced movement of the peak to the right in cells stimulated with XK-81 20 μΜ for 5 h, meaning increased ROS generation (Figure 5A). Furthermore, the exact source for ROS was detected. The results showed that mitochondrial respiratory chain complex I inhibitor rotenone (Rot), rather than NADPH oxidase inhibitor diphenyleneiodonium chloride (DPI), XO inhibitor allopurinol (ALL), mitochondrial respiratory chain complex II inhibitor thenoyltrifluoroacetone (TTFA), and complex III inhibitor antimycin A (AA), regulated the shift of the peak from right to left, highlighting mitochondrial participation (Figure 5B). Furthermore, as shown in Figure 5C, XK-81 killed nearly two-thirds of 4T-1, while ROS scavenger NAC improved the survival rate of 4T-1 cells to 84.62%, indicating the participation of ROS in the process of XK-81-inducing 4T-1 cells’ death.

### 2.6. Ferroptosis Occurred in a XK-81-Treated Mouse Tumor

**Figure 6:**
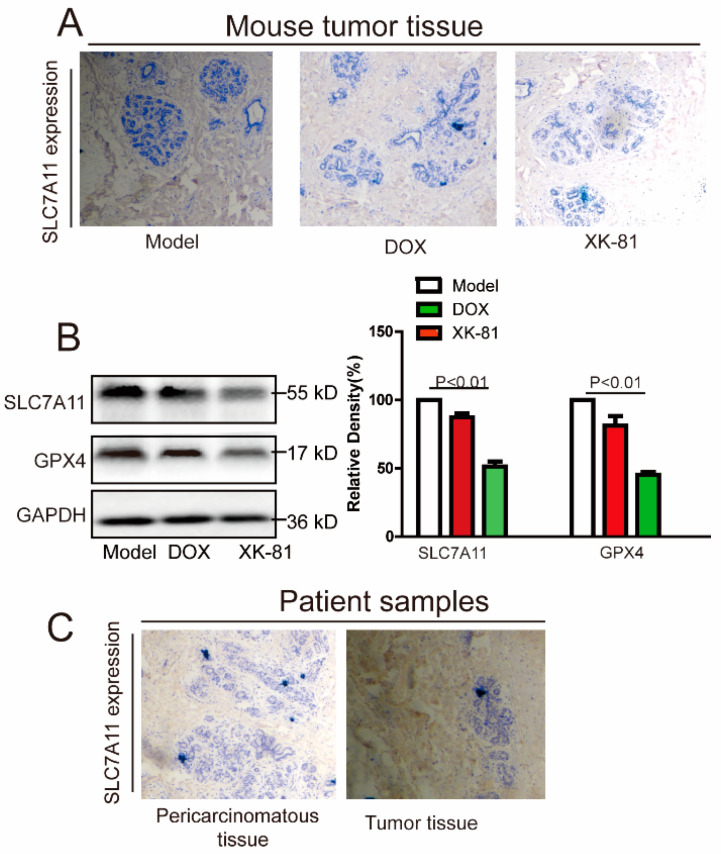
Ferroptosis occurred in XK-81-treated tumor-bearing BALB/C mice. (**A**) Ferroptosis marker SLC7A11 in mice tumor issues was detected with immunohistochemistry. (**B**) After proteins were isolated from tumor tissues, SLC7A11 and GPX4 were detected by Western blotting, and the quantification analysis was performed with Image J. (**C**) The immunohistochemical analysis of the clinical tumor samples was performed, and the expression of SLC7A11 was detected in both tumor tissue and the pericarcinomatous tissue. DOX, doxorubicin; GPX4, glutathione peroxidase 4; XK-81, *Leathesia nana* bromophenol.

To further verify the occurrence of ferroptosis, tumor tissues of mice on the 15th day were collected for immunohistochemical staining and Western blotting after XK-81 administration. Compared with the Model group and the DOX group, the expression levels of ferroptosis inhibiting factor SLC7A11 and GPX4 decreased by nearly one half in the XK-81 group (*p* < 0.01) (Figure 6A,B). These data were consistent with those in vitro. Simultaneously, tumor samples were collected from breast cancer patients for further study. Data from immunohistochemistry assay showed that ferroptosis inhibitor protein SLC7A11 was highly expressed in breast cancer tissues compared with that in pericarcinomatous tissues (Figure 6C). Therefore, we came to a preliminary conclusion that XK-81 induced the death of 4T-1 cells by causing the occurrence of ferroptosis. 

### 2.7. Immunomodulatory Activity of XK-81 in the 4T-1 Mouse Tumor Model

**Figure 7:**
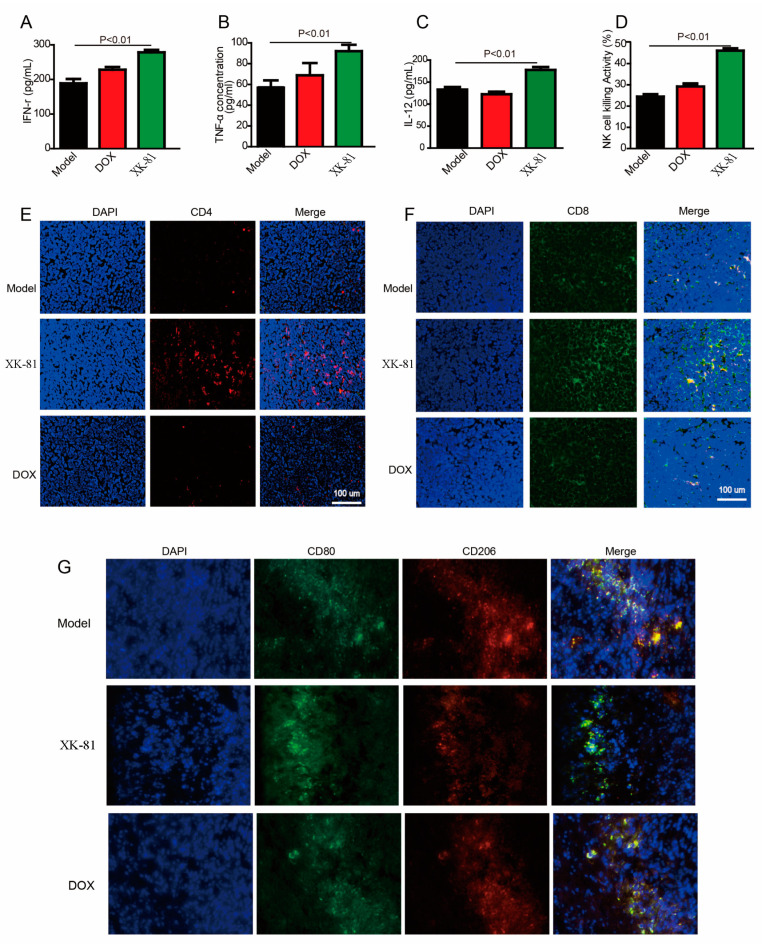
Immunomodulatory activity of XK-81 in treating the 4T-1 mouse tumor. After 14 days of treatment, serum was collected for exam: (**A**) IFN-γ, (**B**) TNF-α, and (**C**) IL-12. (**D**) Mice spleens were isolated, and the activity of NK cells was detected. (**G**) Tumor tissues were isolated and stained for CD80 (green color) to locate M1 macrophages and CD206 (red color) to locate M2 macrophages. (**E**,**F**) Tumor tissues were stained for CD4 (red color) and CD8 (green color) to evaluate CD8+/CD4+ T-cell immune response; DOX, doxorubicin; XK-81, *Leathesia nana* bromophenol; NK cells, natural killer cells.

In the in vivo experiment, serum immune factors IFN-γ (198.34 pg/mL), IL-12 (132.67 pg/mL), and TNF-α (58.89 pg/mL) were detected in serum isolated from model mice. Obviously, the levels of above secretions were apparently elevated in the XK-81 group treatment. As shown in Figure 7A–D, concentrations of IFN-γ, IL-12, and TNF-α were added up to 278.92 pg/mL, 178.95 pg/mL, and 73.58 pg/mL, respectively, exhibiting XK-81’s immunomodulatory effects on 4T1 tumor-bearing mice. Nowadays, tumor-associated macrophages are considered the most abundant innate immune cells in the microenvironment and play a principal role in tumor immune regulation. NK cells also represent an essential component of innate immunity and contribute to anti-tumor immune response. In the following study, NK cell killing activity, CD4+/CD8+ cell proliferation, and the ratio of M1/M2 were detected in tumor tissues. Data from Figure 7D revealed that, compared with model mice, NK cell killing activity was increased up to 43.56%. Moreover, as shown in Figure 7E,F, the XK-81 group showed an exceptionally high percentage of CD8+ (dyed in green) and CD4+ cells (dyed in red) compared with the model group and the DOX group. Furthermore, a large number of tumor-promoting M2 phenotype macrophages (marked with CD206) occurred with red fluorescence in tumor tissues from model mice, while a dominance of tumor-resistant M1 phenotype macrophages (marked with CD80) appeared with green fluorescence in the tissues from XK-81 mice (Figure 7G). The above data evidenced the immunomodulatory activity of XK-81 in treating breast cancer.

### 2.8. XK-81 Activated APCs In Vitro

**Figure 8:**
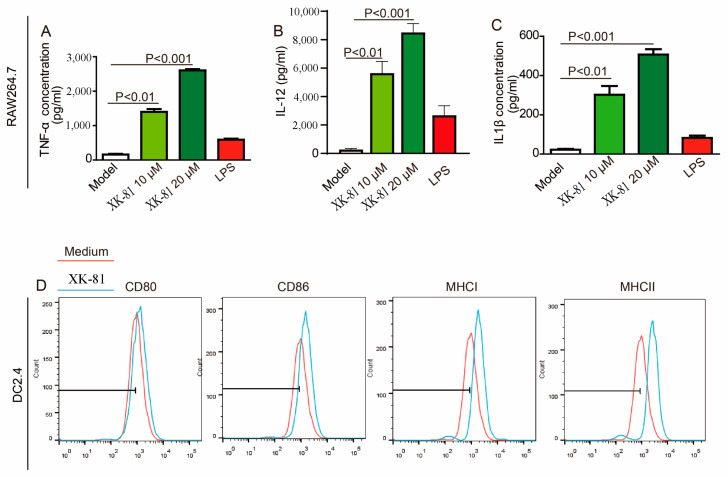
XK-81 activated APCs in vitro. After XK-87 (10 µM, 20 µM) and LPS (1 μg/mL) stimulation for 48 h, respectively, the supernatants of RAW 264.7 cells were collected. The levels of (**A**) TNF-α, (**B**) IL-12, and (**C**) IL-1β in supernatant were detected using ELISA kits; (**D**) After 4T-1 cells were incubated with XK-87 (20 µM) for 48 h, the supernatant was collected and used to stimulate DC2.4 for another 48 h continually. Then, mature marks of DC, including CD80, CD86, MHCI, and MHCII were detected with a flow cytometer. DOX, doxorubicin; XK-81, *Leathesia nana* bromophenol; LPS, Lipopolysaccharide.

DCs are the most potent APCs to induce the proliferation of T cells. They are the master regulators of the immune response and serve this function by linking the microbial sensing features of the innate immune system. Besides macrophages, another important type of cell is the APC cell, which also plays an important role in the activation and the survival of T cells. Figure 8A shows that XK-81 enhanced TNF-α production in a dose-dependent manner in macrophages with the highest level (2578.34 pg/mL), which was achieved with XK-81-20 μM, which was significantly higher than that in the LPS group. A similar result was also obtained in the IL-12 and the IL-1β generations. These findings suggest that XK-81 treatment exhibits immunomodulatory effects on RAW264.7 cells by up-regulating the TNF-α, IL-12, and IL-1β levels. Additionally, the expression levels of antigen complexes (MHCI and MHCII) and co-stimulatory molecules (CD80 and CD86) were detected, respectively [39]. As shown in Figure 8D, compared with the medium group, the expressions of these four types of markers in the DC2.4 cells were upregulated in the XK-81 group. An increase in expression levels of the markers studied would provide an indication of the degree of activation of DC2.4 cells and serve to facilitate the ligand–receptor interactions with effector T cells for increasing the proliferation and the trafficking to tumor sites. The above data showed that XK-81 activated APCs in vitro.

## 3. Materials and Methods

### 3.1. Materials and Chemical Reagents

The novel marine bromophenol compound, XK-81 (Purity ≥ 98%), was supported by the Institute of Oceanology of the Chinese Academy of Sciences (Qingdao, China). Roswell Park Memorial Institute 1640 (RPMI1640), Dulbecco’s modified Eagle’s medium (DMEM), and fetal bovine serum (FBS) were purchased from Life Technologies/Gibco Laboratories (Grand Island, NY, USA); erythrocyte lysis buffer was purchased from Gefan Biotechnology (Shanghai Gefan Biotechnology, Shanghai, China); the Mito-FerroGreen kit was purchased from Dojindo (Kumamoto, Japan); C11 BODIPY 581/591 were purchased from Invitrogen (Camarillo, CA, USA); an immunohistochemistry detection system kit was bought from Bioss (Beijing, China); antibodies for GAPDH, CD80, CD206, GPX4, SLC7A11, and ACSL4 were purchased from ABclonal Technology (Wuhan, China). ELISA kits for IL-12, IL-1β, TNF-α, and IFN-γ were obtained from Nanjing Jiancheng Bioengineering Research Institute (Nanjing, China); all other chemicals were purchased from Sigma Aldrich (St. Louis, MI, USA).

### 3.2. Cell Culture 

Mouse (4T-1, NF639, and EO771) and human (MDA-MB-231 and MCF-7) breast cancer cell lines were purchased from the American Type Culture Collection (Manassas, VA, USA) and cultured in RPMI1640 supplemented with 10% FBS and 1% penicillin/streptomycin, and RAW 264.7 (Manassas, VA, USA) cells were cultured in DMEM supplemented with 10% FBS. All cells were cultured at 37 °C in a humidified atmosphere with 5% CO_2_, as previously described [40].

### 3.3. Cell Viability Assay

XK-81 was dissolved in DMSO, and the final concentration of DMSO was less than 0.1% *v*/*v*, which does not have any other effect on cell activity. The solubility of XK-81 in the tissue culture media was good, with no precipitation or turbidity. 4T-1, NF639, EO771, MDA-MB-231, and MCF-7 cells (1 × 10^4^/well) were seeded in 96-well plates, treated with XK-81 at 5, 10, 15, and 20 µM concentrations for 0 h, 12 h, 24 h, and 48 h, respectively. Additionally, then the diluted MTT solution was added, and the reaction lasted for 4 h. The culture medium was removed, and DMSO was added to dissolve the reaction, forming formazan, and the absorbance was measured immediately after blending [41].

### 3.4. Ethics Statement

All procedures were performed in strict accordance with the NIH guidelines for the care and use of laboratory animals (NIH Publication No. 8023, revised 1978) and followed the regulations for the Care and Use of Laboratory Animals of the National Institute of Animal Health and the Guidance by the ethics committee of Qingdao University (animal welfare assurance number: 14-0027).

### 3.5. Establishment of Breast Cancer Zebrafish Model

The male and female zebrafish were placed in the mating aquarium in a ratio of 1:2 and separated by a partition. The next morning, the partition was removed to start mating, and 30 min later, the embryos were collected. 4T-1 cells were labeled with CM-DiL for 15 min. At an amount of time 48 h post fertilization (48 h pf), zebrafish were anesthetized with 0.003% tricaine. Subsequently, CM-DiL (7.5 μL/mL) labeled 4T-1 cells suspension was injected under a microscope into the yolk sac of 48 h pf zebrafish embryos. After washing with sterile water, they were randomly divided into Model group, XK-81 (2 μM) group, and doxorubicin (DOX, 500 nM) group, and they were treated in an incubator at 28 °C for 48 h. Then fluorescence microscopy was applied to observe the fluorescence intensity [42].

### 3.6. Animal Model and Treatment Protocols

BALB/C mice (female, 6–8 weeks old, 18–22 g) were purchased from Weitonggilhua Co., LTD (Beijing, China). All mice were tested after one week of adaptive feeding. A subcutaneous 4T-1 tumor model was established by inoculating 1 × 10^6^ 4T-1 cells (in 100 µL PBS) (subcutaneous injection) into the right armpit of female BALB/C mice. When the 4T-1 tumor grew to ≈50 mm^3^, mice were randomly divided into 3 groups: the Model group (without drug treatment); the DOX group (3 mg/kg, tail vein injection); and the XK-81 group (50 mg/kg, intraperitoneal injection, dissolved in 0.5% carboxymethyl cellulose-Na). Additionally, they were given drugs every other day. Finally, the mice were killed on the 15th day, and tumor tissues were collected. The tumor volume and body weight of mice were measured every 2 days [43]. The tumor volume was calculated as follows: V = (length) × (width)^2^/2. The efficacy of solid tumors was expressed as tumor weight inhibition percentage (tumor growth inhibition rate): tumor growth inhibition rate = [1−RTV (experimental group)/RTV (control group)] (1−T/C) × 100%; RTV = V_t_/V_0_, V_t_: the tumor volume at the end of the experiment, V_0_: the tumor volume at the beginning of the experiment. At the end of treatment, blood was sampled from rat eyes, hearts, and spleens, which were also isolated for related experiments.

### 3.7. Evaluation of Blood Parameters

Blood samples were collected, and serum samples were separated by centrifugation at 4500 rpm at 4 °C for 20 min after the experiment ended. The serum samples were stored at −80 °C until analysis. The levels of IL-1β, TNF-α, IL-12, and IFN-γ were measured with related biochemical kits.

### 3.8. Preparation and Activity Assay of NK Cells from Spleen

After 14 days of medication, mice were sacrificed, and their spleens were removed in a sterile environment. Spleens were weighed and gently crushed in 3 mL PBS, standing for 5 min. After screening with 200 mesh, the cell suspension was centrifuged at 1500 rpm for 5 min. Erythrocyte lysis buffer was added to the cell pellet to remove erythrocytes. Cell pellets were resuspended by PBS, centrifuged at 1500 rpm for 5 min, and resuspended in complete DMEM medium, adding 1% penicillin/streptomycin and 10% (*v*/*v*) FBS [44]. To assess natural killer (NK) cell activity after they were isolated from splenocyte suspension, YAC-1, the lysis of the NK cell-specific target cell was measured by quantifying the release of lactate dehydrogenase (LDH). After NK cells were co-incubated with YAC-1 cells for 4 h, 100 µL of supernatant was taken into the new well, and 100 µL of LDH conjugate was added and incubated for 15 min. The reaction was terminated after adding 30 µL HCl (1 M) per well. Finally, optical density was measured at 490 nm.

### 3.9. Echocardiography

After 14 days of medication, a high-resolution ultrasound imaging system (Vevo2100; VisualSonics, Inc, Toronto, ONT (Ontario), Canada), equipped with a 30-MHz mechanical transducer, was applied to detect transthoracic echocardiography. After mice were lightly anesthetized with 4% paraformaldehyde and placed on a warming platform (37 °C), heart function, including ejection fraction (EF), fractional shortening (FS), left ventricular end-diastole volume (LVEDV), and left ventricular end-systolic volume (LVESV) were measured by two-dimensional guided M-mode echocardiography.

### 3.10. Identification of Hallmarks of Ferroptosis 

To detect Fe^2+^ ion, treated 4T-1 cells from 24-well plates were stained with 1 µM FerroGreen for 30 min in the dark at 37 °C. Then fluorescence intensity was visualized with an inverted fluorescence microscope. Besides, 4T-1s seeded in 6-well plates were stained with C11 BODIPY 581/591 10 µM for 30 min in the dark and then collected by centrifugation at 1500 rpm for 5 min. Finally, the flow cytometry (ThermoFisher Scientific, Waltham, MA, USA) was applied to detect the lipid peroxidation level.

### 3.11. Clinical Samples

The procedures were approved by the ethics committee of Qingdao University (Qingdao, China), and informed consent was obtained from all patients. We obtained tumors tissues and pericarcinomatous tissues from 56 breast cancer patients from The Affiliated Hospital of Qingdao University (Qingdao, China) and stored them at −80 °C until use.

### 3.12. Determination of Reactive Oxygen Species (ROS) Generation

After XK-81 treatment for 1 h, 3 h, and 5 h respectively, the 4T-1 cells were washed with PBS. DCFH-DA (10 µM) was added and incubated in a cell culture box at 37 °C for 20 min, according to the reagent specification’s instructions. 4T-1 cells were collected after PBS washing and trypsin digestion. Then, the fluorescence intensity of the cells was measured by low cytometry (ThermoFisher Scientific) [45].

### 3.13. Immunohistochemistry Analysis

The tumor tissue was sequentially sectioned after fixation with paraformaldehyde and paraffin-embedded, and then it was dewaxed and hydrated in xylene and gradient ethanol. An amount of 0.01 M sodium citrate was used for antigen repair in a microwave. The sections were then treated with 3% hydrogen peroxide in methanol, and then they were blocked with 5% bovine serum albumin (BSA) to block non-specific binding. Then, the sections were incubated with a primary antibody (SLC7A11, 1:100) overnight at 4 °C. After the second antibody was incubated, a chromogenic agent was added, hematoxylin was redyed, and the tablets could be sealed and observed under a microscope after gradient ethanol dehydration [46].

### 3.14. Western Blotting Analysis

As previously described [47], cells were washed with precooled PBS. RIPA protein lysate was added, and then the related proteins were obtained after heating and deformation for protein electrophoresis. Equivalent protein amounts were transferred to the PVDF membrane. After the PVDF membrane was sealed in skim milk powder for 1 h, it was incubated with primary antibodies (GPX4-1:1000, SLC7A11-1:1000, ACSL4-1:1000, CD8α-1:1000) at 4 °C overnight. Whereafter, a HRP-linked antibody was incubated at room temperature for 1 h. Finally, drop ECL chemiluminescence solution was added, and chemiluminescence signals were detected with ChemiDocTM Imager. Considering that the MW ranges of membrane strips are close (GAPDH: 36kD, SLC7A11:55kD, GPX4:17kD; ACSL4:79kD), to keep the circumstances as consistent as possible to reduce the influence of external factors that might create variation in the results, we detected all the protein expressions in the same strip. So, the membranes were stripped and incubated again. The related experimental procedures were as follows. Firstly, rinse membranes with water to remove chemiluminescent substrate. Then, incubate the membrane in the stripping buffer (0.5M Tris HCL, Ph6.8, 12.5Ml; 10% SDS, 20 mL; 2-mercaptoethanol, 0.8 mL; deionized water, 67.5 mL) for 20 min at room temperature fiercely. After washing the membranes 3 times with agitation for 5 min in PBST, incubate the membranes with milk overnight. Finally, reapply primary and secondary antibodies. The order for adding primary antibody is: ACSL4—SLC7A11—GPX4—GAPDH. Besides, membranes were cut horizontally to separate the high molecular weight membrane (ACSL4) and the low ones (SLC7A11, GAPDH and GPX4).

### 3.15. Flow Cytometry Analysis 

To detect the effect of XP-81 on APCs, DC2.4 cell lines were treated in vitro, as previously described [48]. Firstly, 4T-1 cells were incubated with XP-81 for 48 h, then the supernatant was extracted to stimulate DC2.4. After 48 h, the cell surfaces were labeled with PE anti-mouse CD80, PE anti-mouse CD86, PE anti-mouse MHC I, and PE anti-mouse MHC II at 4 °C for 30 min. Flow cytometry was performed on a BD LSR II (BD Biosciences, San Jose, CA, USA) and analyzed using FlowJo v10.6.2 software (Tree Star, San Carlos, CA, USA). Each experiment was performed in triplicate. 

### 3.16. Immunofluorescence Analysis

The frozen section of the tumor tissue samples from model group, DOX group, and XP-81 group were incubated with antibodies CD80, CD4, CD8, and CD206 at 4 °C overnight. Then, the sections were sealed with fluorescence secondary antibody containing 4′,6-diamidino-2-phenylindole (DAPI) for 4 h at room temperature. Finally, a fluorescence microscope (Nikon A1 MP, Tokyo, Japan) was used to examine the slides. Quantitative analysis was performed using Image J V1.8.0.112 software [49].

### 3.17. Statistical Analysis

All assays were carried out at least in triplicate, and the results were reported as means ± standard deviation (SD) or standard error of mean (SEM). Statistical comparisons were performed by using one-way ANOVA analysis using the SPSS 19.0 software. Data significance was set at *p* < 0.01.

## 4. Conclusions

**Figure 9:**
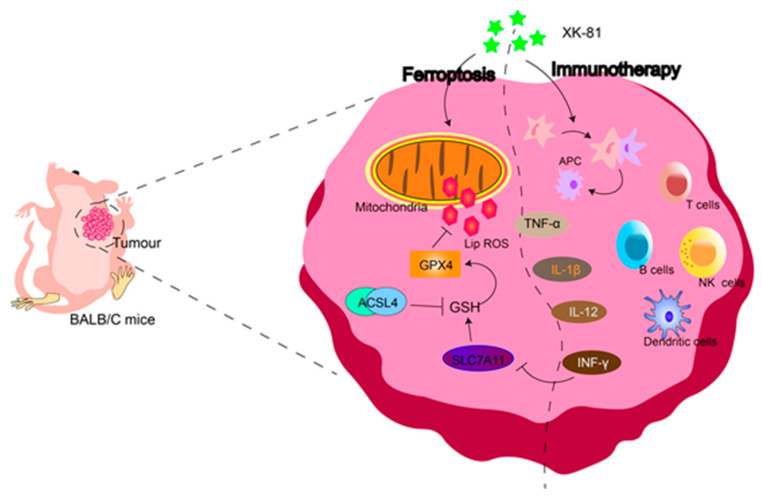
XK-81 has potential and advantages for treating breast cancer by combining ferroptosis and immunotherapy.

In summary, our data demonstrated that XK-81, a novel bromophenol compound, has potential and advantages for treating breast cancer by combining ferroptosis and immunotherapy (Figure 9). Our study offered an ideal anti-cancer candidate from the ocean and also sought a feasible strategy in the future.

## Data Availability

Not applicable.
